# One-step triplex TaqMan quantitative reverse transcription polymerase chain reaction for the detection of feline coronavirus, feline panleukopenia virus, and feline leukemia virus

**DOI:** 10.14202/vetworld.2024.946-955

**Published:** 2024-05-04

**Authors:** Mengyi He, Shuping Feng, Kaichuang Shi, Yandi Shi, Feng Long, Yanwen Yin, Zongqiang Li

**Affiliations:** 1 Department of Basic Veterinary Medicine, College of Animal Science and Technology, Guangxi University, Nanning 530005, China; 2 Guangxi Center for Animal Disease Control and Prevention, Nanning 530001, China

**Keywords:** detection method, feline coronavirus, feline leukemia virus, feline panleukopenia virus, multiplex reverse transcription-quantitative polymerase chain reaction

## Abstract

**Background and Aim:**

Feline coronavirus (FCoV), feline panleukopenia virus (FPV), and feline leukemia virus (FeLV) are prevalent throughout China and significantly threaten cat health. These viruses cause similar manifestations and pathological damage. Rapid and accurate diagnosis depends on detection in the laboratory. This study aimed to establish a reliable and rapid method for accurate detection of FCoV, FPV, and FeLV so that a definite diagnosis can be made and effective measures can be taken to prevent and control viral infection.

**Materials and Methods:**

We designed three pairs of specific primers and probes for the detection of FCoV 5′ untranslated region, FPV viral protein 2, and FeLV pol genes. Recombinant plasmid constructs were generated for use as standard plasmid constructs. Optimal reaction conditions, including primer and probe concentrations, reaction cycles, and annealing temperatures, were obtained on the basis of optimization tests. One-step triplex real-time reverse transcription-quantitative polymerase chain reaction (RT-qPCR) was successfully established to simultaneously detect FCoV, FPV, and FeLV. The specificity, sensitivity, and repeatability of the assay were analyzed, and its applicability was validated by testing 1175 clinical samples.

**Results:**

One-step triplex RT-qPCR had a high degree of specificity only for the detection of FCoV, FPV, and FeLV; it had high sensitivity with limits of detection of 139.904, 143.099, and 152.079 copies/reaction for p-FCoV, p-FPV, and p-FeLV standard plasmid constructs, respectively, and it had reliable repeatability with 0.06%–0.87% intra-assay coefficients of variations. A total of 1175 clinical samples were examined for FCoV, FPV, and FeLV using triplex RT-qPCR, and the FCoV, FPV, and FeLV positivity rates were 18.47%, 19.91%, and 47.57%, respectively. The clinical sensitivity and specificity of one-step triplex RT-qPCR were 93.07% and 97.99%, respectively.

**Conclusion:**

We developed a rapid and reliable one-step triplex RT-qPCR method for the detection of FCoV, FPV, and FeLV, which could be used as a diagnostic tool for clinical monitoring and diagnosis.

## Introduction

In view of their close contact with humans, the health issues of companion animals have attracted considerable attention. Three common viruses, feline coronavirus (FCoV), feline panleukopenia virus (FPV), and feline leukemia virus (FeLV), are prevalent throughout China and significantly jeopardize cat health.

FCoV is an enveloped, single-stranded, and positive-sense RNA virus belonging to *Alphacoronavirus* of the *Coronaviridae* family [1]. The viral genome (approximately 29 kb) contains 11 open reading frames, which encode four structural proteins and seven non-structural (NS) proteins. FCoV is divided into two biotypes based on its pathogenicity: feline enteric coronavirus (FECV) and feline infectious peritonitis virus (FIPV) [2]. FECV infection is mainly limited to the intestine, leading to mild, self-limiting gastrointestinal diseases. FIPV causes a deadly, multi-system, immune-mediated disease that damages a variety of tissues and organs, and peritonitis or even death is the most typical sign of damage [2, 3]. FIPV is considered to be a mutant of FECV, resulting in changes in viral pathogenicity and tropism. However, the genetic differences that can explain the different pathogenicities of FECV and FIPV remain unclear [1, 4, 5]. FCoV can be divided into serotype I and serotype II according to viral antigenic differences, and serotype II virus arises from two-fold homologous recombination between serotype I FCoV and serotype II canine coronavirus [6, 7]. At present, Type I and Type II FCoVs are prevalent in many countries around the world [8–10]. In China, Type I FCoV and Type II FCoV are simultaneously prevalent, with Type I FCoV being the main serotype [11–14].

FPV, a member of the genus *Protoparvovirus* in the family *Parvoviridae*, is a non-enveloped, single-stranded DNA virus [15]. FPV contains a 5.1 kb genome that encodes NS and structural proteins. Clinical manifestations include depression, fever, vomiting, diarrhea, and high dehydration. The main characteristic is a sharp reduction of white blood cell count, which leads to death in severe cases [16, 17].

FeLV, a member of the *Gammaretrovirus* genus in the family *Retroviridae*, is an enveloped, single-stranded, positive-sense RNA virus. FeLV is a typical γ retrovirus with a genome that is converted into proviral DNA and integrated into the host cell’s native genetic material [18]. FeLV-infected animals show or do not show clinical symptoms at different stages of infection depending on the immune status and infection pressure [19]. Clinical signs of FeLV infection include lymphatic tumors, immunosuppression, hematological conditions, and reproductive issues [18, 19]. Polymerase chain reaction (PCR) can detect viral RNA or provirus, and detection of proviral DNA is usually an ideal choice [20, 21].

FCoV [8–14], FPV [22–26], and FeLV [27–29] are prevalent in China and other countries worldwide, and coinfection with these two and/or three viruses is common [8, 30–32], resulting in huge economic damage and increasing health-care attention. FCoV, FPV, and FeLV cause similar signs of depression, vomiting, diarrhea, dehydration, and weight loss [1, 3, 16, 18, 19], making it difficult to differential diagnosis of these diseases based only on clinical manifestations. It is vital to develop a specific, sensitive, and rapid detection method to accurately diagnose and rapidly propose effective measures for these diseases. TaqMan quantitative PCR (qPCR) has the advantages of high throughput, being sensitive, specific, and uneasy to contamination and has been widely applied in different laboratories for the detection of viral nucleic acids [33]. To date, singleplex and multiplex reverse transcription-qPCR (RT-qPCR)/qPCR have been described for detecting FCoV, FPV, and/or FeLV [16, 34–37]. However, no multiplex RT-qPCR for simultaneous detection and differentiation of FCoV, FPV, and FeLV has been reported till date.

This study aimed to develop a one-step triplex RT-qPCR based on the TaqMan probe for the simultaneous detection of FCoV, FPV, and FeLV, and to evaluate the applicability of this method in clinical samples. One-step triplex RT-qPCR could rapidly and accurately detect and differentiate FCoV, FPV, and FeLV with one reaction in one tube.

## Materials and Methods

### Ethical approval

No live cats were used in this study. All feces and anal and nasal swabs were collected from different veterinary hospitals. Ethical approval was not necessary for this study, as per the guidelines of the Guangxi Center for Animal Disease Control and Prevention (CADC), China.

### Study period and location

This study was conducted from September 1, 2021 to December 31, 2023 in Guangxi CADC, China.

### Virus strains and clinical samples

The vaccine strains were purchased from Zoetis Enterprise Management (Shanghai, China) Co., Ltd., including the Cu-4 strain of FPV, the 605 strain of feline herpesvirus (FHV), and the 255 strain of feline calicivirus (FCV). FCoV, FeLV, feline bocavirus (FBoV), and feline astrovirus (FeAstV) were positive clinical samples that were verified by PCR/RT-PCR and gene sequencing and were provided by Guangxi CADC, China.

A total of 1175 clinical samples, including feces and anal and nasal swabs from healthy and sick cats, were collected from different veterinary hospitals in Guangxi province from 2021 to 2023 and stored at –80°C until use.

### Extraction of nucleic acids

The positive clinical samples of FCoV and FeLV were treated with phosphate-buffered saline (PBS; pH 7.2, W/V = 1:5), vortexed for 30 s, and 200 μL supernatants were used to exact total RNA using a DNA/RNA extraction kit (Tianlong, Xi’an, China) according to the manufacturer’s instructions. The extracted RNA was reverse transcribed into cDNA by Oligo(dT) using the PrimeScript™ II 1^st^ Strand cDNA synthesis kit (TaKaRa, Dalian, China) according to the manufacturer’s instructions. Two hundred microliters of the Cu-4 vaccine strain of FPV solution were used to exact total DNA using a DNA/RNA extraction kit (Tianlong, Xi’an) according to the manufacturer’s instructions. All DNA and cDNA samples were stored at –80°C until they were used to generate the recombinant standard plasmid construct.

Two hundred microliters of the vaccine solution of FHV and FCV or 200 μL supernatants of the positive clinical samples of FBoV and FeAstV (after treatment with PBS and vortex) were used to extract total DNA/RNA using a DNA/RNA extraction kit (Tianlong, Xi’an) according to the manufacturer’s instructions and to analyze the specificity of the developed one-step triplex RT-qPCR in this study.

The 1175 clinical samples were treated with PBS (pH 7.2, W/V = 1:5), vortexed for 30 s, and 200 μL supernatants of each sample were used to exact total DNA/RNA using a DNA/RNA extraction kit (Tianlong, Xi’an) according to the manufacturer’s instructions. Total DNA/RNA was used to test FCoV, FPV, and FeLV using the developed one-step triplex RT-qPCR in this study, in which the downstream primers were used to reverse-transcribe the RNA templates.

### Design of specific primers and probes

The genomic sequences of the representative FCoV, FPV, and FeLV strains were downloaded from National Center for Biotechnology Information GenBank (https://www.ncbi.nlm.nih.gov/nucleotide/, accession on November 16, 2021), aligned, and compared. Three pairs of primers and probes target the conserved regions of the 5′ untranslated region (UTR) of FCoV, the viral protein 2 (VP2) gene of FPV, and the pol gene of FeLV (Table-1). These primers and probes were simultaneously used to detect different types and strains of FCoV, FPV, and FeLV. Supplementary Figure-1 shows multiple nucleotide alignments of the reference sequences and the primer and probe locations. The specificity of the primers and probes was confirmed by GenBank blast analysis (https://blast.ncbi.nlm.nih.gov/Blast.cgi, accession on November 16, 2021).

**Table 1:** Primer and probe sequences of the triplex RT-qPCR.

Name	Sequence (5′→3′)	Tm/°C	Gene	Product/bp
FCoV-F	CCTGTTTGGTAAGTCGTCTAGTAT	57.6	5′UTR	130
FCoV-R	CGAGGATCTTAAATTGTTTGGAACT	57.5		
FCoV-P	FAM-TAGTTGGGTAGACCGGGTTCCGTC-BHQ1	65.2		
FPV-F	GCTACTCAGCCACCAACTAAA	56.3	VP2	103
FPV-R	TCATAGCTGCTGGAGTAAATGG	57.5		
FPV-P	CY5-ACTGCATCATTGATGGTTGCATTAG-BHQ3	60.8		
FeLV-F	AATGGACCCGCCTTTATCTC	57.0	pol	108
FeLV-R	TTCTACCTGACCTGAACTTTGG	54.9		
FeLV-P	VIC-TAAGTCAGTCTGTGGCCACCCTACT-BHQ1	65.5		

FCoV=Feline coronavirus, FPV=Feline panleukopenia virus, FeLV=Feline leukemia virus, RT-qPCR=Reverse transcription-quantitative polymerase chain reaction

**Figure-1:**
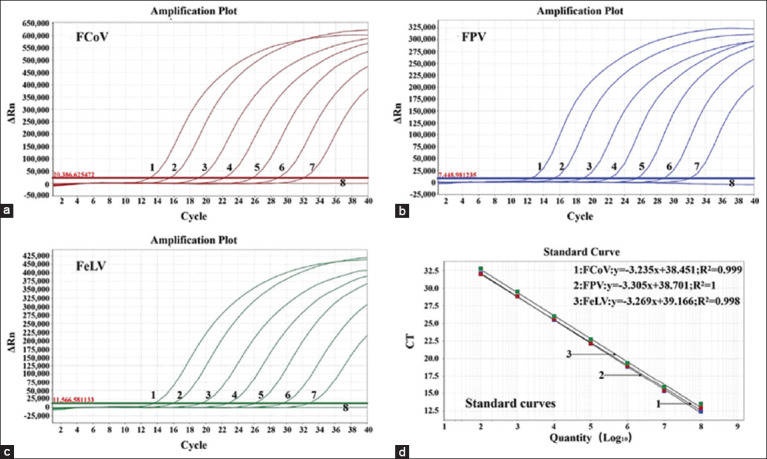
The amplification curves (a-c) and standard curves (d). Amplification curves were generated using the standard plasmid constructs p-FCoV (a), p-FPV (b), and p-FeLV (c) with final reaction concentrations of 1.50 × 10^7^–1.50 × 10^1^ copies/μL. The standard curves (d) showed a good linear relationship (R^2^ ≥ 0.998). FCoV=Feline coronavirus, FPV=Feline panleukopenia virus, FeLV=Feline leukemia virus.

### Generation of standard plasmids

The targeted fragments of FCoV, FPV, and FeLV were amplified by PCR using the obtained DNA and cDNA templates. The reaction system contains 12.5 μL of 2× Premix Taq, 0.4 μL (25 pmol/μL) of forward and reverse primers, 2.5 μL of template, and nuclease-free distilled water to make a total volume of 25 μL. The amplification procedure included 94°C for 3 min, 35 cycles of 94°C for 30 s, 55°C for 30 s, 72°C for 10 s, and 72°C for 10 min.

The PCR products were separated using 1.5% Agarose gel electrophoresis, purified using the MiniBEST DNA fragment purification kit Ver.4.0 (TaKaRa, Dalian, China), connected to the pMD18-T vector (TaKaRa, Dalian, China), and transformed into *Escherichia coli* DH5α competent cells (TaKaRa, Dalian, China) according to the manufacturer’s instructions. The positive clones were cultured overnight (22–24 h) at 37°C, and the plasmid was extracted using the MiniBEST plasmid purification kit Ver.4.0 (TaKaRa, Dalian, China). The plasmid concentration was calculated according to the absorbance measurements at optical density (OD)_260nm_ and OD_280nm_. The three recombinant plasmid constructs were named p-FCoV, p-FPV, and p-FeLV and were used to establish one-step triplex RT-qPCR. The formula for the concentration calculation of the plasmid constructs was as follows:


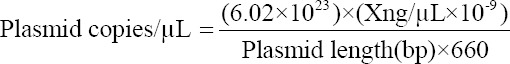


### Evaluation of the reaction conditions

All experiments were performed using the QuantStudio 5 qPCR detection system (ABI, Carlsbad, CA, USA). The total volume of the reaction system was 20 μL. Different annealing temperatures (55°C–60°C), primer and probe concentrations (0.2–0.5 μL, 20 pmol/μL), and reaction cycles (25–45 cycles) were arranged and combined to determine the optimal reaction system and parameters. The reaction system comprised 10 μL 2× One-Step RT-PCR buffer III (TaKaRa, Dalian, China), 0.4 μL Ex Taq HS (5 U/μL), 0.4 μL PrimeScript^RT^ enzyme mix II, three pairs of primers and probes with different concentrations, 2 μL of a mixture of three standard plasmid constructs, and nuclease-free distilled water up to 20 μL. The amplification procedure consisted of 42°C for 5 min, 95°C for 10 s, and 40 cycles of 95°C for 5 s and 55°C for 30 s. Fluorescence signals were automatically collected by the qPCR detection system at the end of each cycle.

### Generation of standard curves

The mixtures of p-FCoV, p-FPV, and p-FeLV plasmid constructs were serially diluted 10-fold from 1.50 × 10^8^ copies/μL to 1.50 × 10^2^ copies/μL and used as templates to generate the standard curves. To draw the standard curves and calculate the standard equations, take the logarithm of the plasmid copy number as the abscissa and the measured Ct value as the ordinate, and determine E values (amplification efficiency), and R^2^ values (correlation coefficient).

### Specificity analysis

To confirm specificity, one-step triplex RT-qPCR was used to detect FCoV, FPV, FeLV, FCV, FHV, FBoV, and FeAstV. Negative controls included negative clinical samples and nuclease-free distilled water.

### Sensitivity analysis

The mixture of plasmid constructs of p-FCoV, p-FPV, and p-FeLV was serially diluted 10-fold to obtain a concentration ranging from 1.50 × 10^7^ to 1.50 × 10^−1^ copies/μL (final reaction concentrations: 1.50 × 10^6^ to 1.50 × 10^−2^ copies/μL), and used as templates for determining the limits of detection (LODs) of the one-step triplex RT-qPCR.

In addition, LODs were obtained by two-fold serial dilution of the mixed plasmid constructs at four different concentrations (500, 250, 125, and 62.5 copies/reaction) and performing 24 repeated experiments using PROBIT regression analysis.

### Repeatability analysis

To assess repeatability, concentrations of 1.50 × 10^7^, 1.50 × 10^5^, and 1.50 × 10^3^ copies/μL were selected. Each concentration of the plasmid constructs was repeated 3 times on 3 different days in each independent experiment. Intra-assay and inter-assay variations were evaluated by calculating coefficients of variations (CVs).

### Detection of clinical samples

All 1175 samples collected from cats in veterinary hospitals in Guangxi province were tested using the established one-step triplex RT-qPCR method, and the positivity rate of each virus was analyzed. In addition, previously reported qPCR methods by Tandon *et al*. [36] and Thieulent *et al*. [38] have been used to test the 1175 clinical samples. We compared the results obtained using these two methods and assessed the clinical sensitivity and specificity.

## Results

### Generation of standard plasmid constructs

Three recombinant plasmid constructs were generated after PCR amplification, purification, ligation, transformation, identification, culture of positive clones, and plasmid extraction. They were named p-FCoV, p-FPV, and p-FeLV and were used as standard plasmid constructs. Initial concentrations were determined to be 2.78 × 10^10^, 2.97 × 10^10^, and 3.24 × 10^10^ copies/μL, respectively, and were then diluted to 1.50 × 10^10^ copies/μL for use.

### Determination of the optimal reaction conditions

The final optimal conditions for one-step triplex RT-qPCR were determined after optimizing the different reaction conditions. Table-2 shows the reaction system with a total volume of 20 μL and the components in detail. The thermocycling conditions were 42°C for 5 min and 95°C for 10 s, followed by 40 cycles of 95°C for 5 s and 55°C for 30 s. Fluorescence signals were recorded at the end of each reaction cycle. Samples with Ct values ≤36 were considered positive samples.

**Table 2:** The composition of the reaction system.

Reagent	Volume/µL	Final concentration/nM
2 × One-Step RT-PCR Buffer III (TaKaRa)	10.0	/
Ex Taq HS (5 U/mL) (TaKaRa)	0.4	/
PrimeScript RT enzyme mix II (TaKaRa)	0.4	/
FCoV (5′UTR)-F (20 pmol/µL)	0.3	300
FCoV (5′UTR)-R (20 pmol/µL)	0.3	300
FCoV (5′UTR)-P (20 pmol/µL)	0.3	300
FPV (VP2)-F (20 pmol/µL)	0.3	300
FPV (VP2)-R (20 pmol/µL)	0.3	300
FPV (VP2)-P (20 pmol/µL)	0.3	300
FeLV (pol)-F (20 pmol/µL)	0.3	300
FeLV (pol)-R (20 pmol/µL)	0.3	300
FeLV (pol)-P (20 pmol/µL)	0.3	300
Nucleic acid template	2.0	/
Nuclease-free distilled H_2_O	Up to 20	/

RT-PCR=Reverse transcription-polymerase chain reaction, FCoV=Feline coronavirus, FPV=Feline panleukopenia virus, FeLV=Feline leukemia virus, 5′UTR=5′ untranslated region, VP2=Viral protein 2

### Generation of standard curves

To generate standard curves for one-step triplex RT-qPCR, mixtures of standard plasmid constructs p-FCoV, p-FPV, and p-FeLV with final reaction concentrations ranging from 1.50 × 10^7^ to 1.50 × 10^1^ copies/μL were used. The slope, correlation (R^2^), and amplification efficiency (E) of the standard curves were determined for FCoV (–3.235, 0.999, 103.756%), FPV (–3.305, 1, 100.707%), and FeLV (–3.269, 0.998, 102.253%) (Figure-1), and all had favorable correlation coefficients and amplification efficiency.

### Specificity analysis

**Figure-2:**
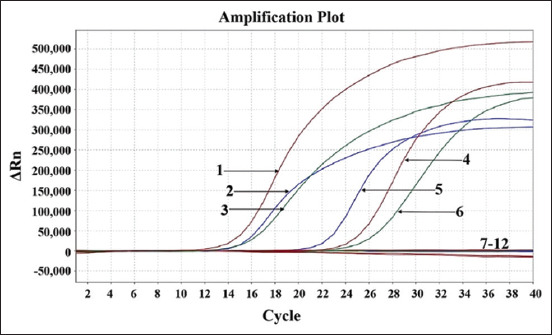
Specificity analysis. 1: p-FCoV; 2: p-FPV; 3: p-FeLV; 4: FCoV; 5: FPV; 6: FeLV; 7-10: FCV, FHV, FBoV, and FeAstV; 11: Negative clinical sample; 12: Nuclease-free distilled water. FCoV=Feline coronavirus, FPV=Feline panleukopenia virus, FeLV=Feline leukemia virus, FCV=Feline calicivirus, FHV=Feline herpesvirus, FBoV=Feline bocavirus, FeAstV=Feline astrovirus.

To evaluate specificity, FCoV, FPV, FeLV, FCV, FHV, FBoV, and FeAstV DNA/RNA were used as templates for one-step triplex RT-qPCR. The results showed that only FCoV, FPV, and FeLV were tested as positive with amplification curves, whereas no amplification curve was observed for the other viruses (Figure-2).

### Sensitivity analysis

**Figure-3:**
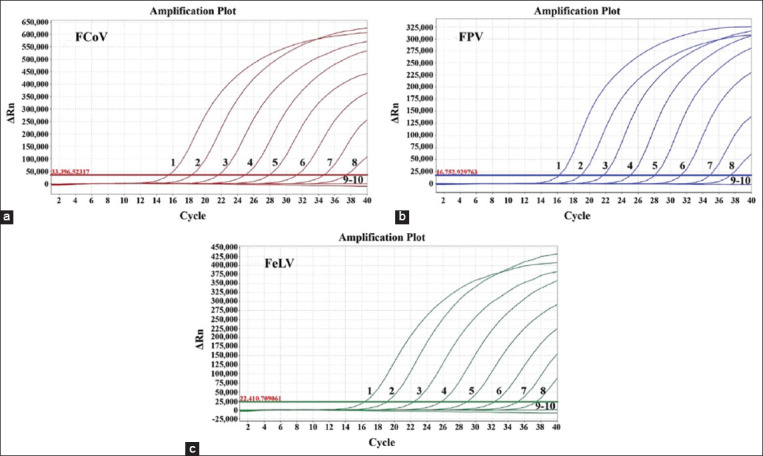
Sensitivity analysis. 1-9: The standard plasmid constructs of (a) p-FCoV, (b) p-FPV, and (c) p-FeLV with final concentration of 1.50 × 10^6^ ~ 1.50 × 10^−2^ copies/μL; 10: Nuclease-free distilled water. The limits of detection of FCoV, FPV, and FeLV were 15 copies/μL. FCoV=Feline coronavirus, FPV=Feline panleukopenia virus, FeLV=Feline leukemia virus.

A mixture of three plasmid constructs with final reaction concentrations from 1.50 × 10^6^ to 1.50 × 10^−2^ copies/μL were used as templates for triplex RT-qPCR to evaluate sensitivity. As shown in Figure-3, the Ct values were <36 when the plasmid constructs were 15 copies/μL, indicating that the LODs of p-FCoV, p-FPV, and p-FeLV for one-step triplex RT-qPCR was 15 copies/μL.

**Figure-4:**
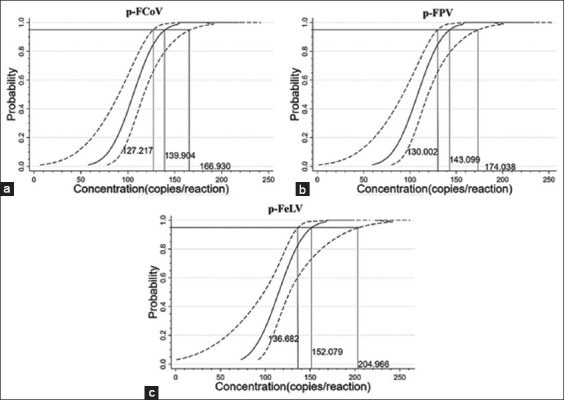
Sensitivity analysis using the Probit regression analysis. The limits of detection for p-FCoV (a), p-FPV (b), and p-FeLV (c) were 139.904 (127.217–166.930 at 95% confidence interval [CI]), 143.099 (130.002–174.038 at 95% CI), and 152.079 (136.682–204.966 at 95% CI) copies/reaction, respectively. FCoV=Feline coronavirus, FPV=Feline panleukopenia virus, FeLV=Feline leukemia virus.

Probit regression analysis was used to analyze the Ct values and hit rates of the different diluted templates (Table-3). LODs for p-FCoV, p-FPV, and p-FeLV were determined to be 139.904 (127.217–166.930 at 95% confidence interval (CI), 143.099 (130.002–174.038 at 95% CI), and 152.079 (136.682–204.966 at 95% CI) copies/reaction, respectively (Figure-4).

**Table 3:** Threshold cycle (Ct) values and hit rates of serially diluted plasmid constructs.

Plasmid	Copies/reaction	Number of samples	Multiplex RT-qPCR	

			Ct (average)	Hit rate (%)
p-FCoV	500	24	33.96	100
	250	24	35.01	100
	125	24	36.03	83.33
	62.5	24	ND	0
p-FPV	500	24	34.08	100
	250	24	35.07	100
	125	24	36.07	79.17
	62.5	24	ND	0
p-FeLV	500	24	34.59	100
	250	24	35.58	100
	125	24	36.55	66.67
	62.5	24	ND	0

FCoV=Feline coronavirus, FPV=Feline panleukopenia virus, FeLV=Feline leukemia virus, RT-qPCR=Reverse transcription-quantitative polymerase chain reaction

### Repeatability analysis

Mixtures of three plasmid constructs with final concentrations of 1.50 × 10^7^, 1.50 × 10^5^, and 1.50 × 10^3^ copies/μL were used for repeatability verification. The intra- and inter-assay CVs were 0.06%–0.87% and 0.40%–1.45%, respectively (Table-4).

**Table 4:** The repeatability of the triplex RT-qPCR.

Plasmid	Concentration (copies/μL)	Ct value of intra-assay			Ct value of inter-assay		
	
		*X̅*	SD	CV (%)	*X̅*	SD	CV (%)
p-FCoV	1.50 × 10^7^	15.46	0.03	0.20	15.38	0.06	0.40
	1.50 × 10^5^	22.21	0.19	0.87	22.44	0.21	0.94
	1.50 × 10^3^	28.61	0.11	0.39	28.50	0.41	1.45
p-FPV	1.50 × 10^7^	15.39	0.12	0.79	15.23	0.14	0.92
	1.50 × 10^5^	21.83	0.14	0.64	22.09	0.29	1.29
	1.50 × 10^3^	28.50	0.19	0.68	28.57	0.41	1.42
p-FeLV	1.50 × 10^7^	15.95	0.06	0.35	15.65	0.21	1.36
	1.50 × 10^5^	22.90	0.16	0.70	22.70	0.25	1.10
	1.50 × 10^3^	29.19	0.02	0.06	29.31	0.27	0.92

FCoV=Feline coronavirus, FPV=Feline panleukopenia virus, FeLV=Feline leukemia virus, RT-qPCR=Reverse transcription-quantitative polymerase chain reaction, SD=Standard deviation, CI=Confidence interval, Ct=Threshold cycle

### Application for clinical sample detection

One-step triplex RT-qPCR was used to evaluate the 1175 clinical samples (Table-5). The FCoV, FPV, and FeLV positivity rates were 18.47% (217/1175), 19.91% (234/1175), and 47.57% (559/1175), respectively. The coinfection rates of FCoV + FPV, FCoV + FeLV, FPV + FeLV, and FCoV + FPV + FeLV were 2.64% (31/1175), 9.62% (113/1175), 7.06% (83/1175), and 1.96% (23/1175), respectively. In addition, 1175 clinical samples were also evaluated using qPCR [36, 38], and the FCoV, FPV, and FeLV positivity rates were 18.55% (218/1175), 19.66% (231/1175), and 47.23% (555/1175), respectively.

**Table 5:** The positivity rate of clinical samples.

Area	Number	Number of positive samples (%)						

		FCoV	FPV	FeLV	FCoV + FPV	FCoV + FeLV	FPV + FeLV	FCoV + FPV + FeLV
Beihai	10	1 (10.00)	4 (40.00)	7 (70.00)	0	0	3 (30.00)	1 (10.00)
Hechi	21	8 (38.09)	20 (95.24)	9 (42.86)	5 (23.81)	0	5 (23.81)	3 (14.28)
Guilin	69	11 (15.94)	18 (26.09)	51 (73.91)	1 (1.45)	8 (11.59)	10 (14.49)	2 (2.90)
Baise	137	33 (24.09)	50 (36.50)	64 (46.71)	5 (3.65)	11 (8.03)	16 (11.68)	7 (5.11)
Qinzhou	15	4 (26.67)	0	10 (66.67)	0	4 (26.67)	0	0
Yulin	264	12 (4.54)	37 (14.01)	8 (3.03)	7 (2.65)	3 (1.14)	1 (0.38)	1 (0.38)
Nanning	338	77 (22.78)	57 (16.86)	243 (71.89)	11 (3.25)	52 (15.38)	27 (7.99)	3 (0.89)
Liuzhou	321	71 (22.12)	48 (14.95)	167 (52.02)	2 (0.62)	35 (10.90)	21 (6.54)	6 (1.87)
Total	1175	217 (18.47)	234 (19.91)	559 (47.57)	31 (2.64)	113 (9.62)	83 (7.06)	23 (1.96)

FCoV=Feline coronavirus, FPV=Feline panleukopenia virus, FeLV=Feline leukemia virus, RT-qPCR=Reverse transcription-quantitative polymerase chain reaction

We compared the detection results of one-step triplex RT-qPCR with those of previously reported methods by Tandon *et al*. [36] and Thieulent *et al*. [38]. The clinical sensitivity and specificity of one-step triplex RT-qPCR for FCoV, FPV, and FeLV were 94.50% and 98.85%, 93.07% and 97.99%, and 99.10% and 98.55%, respectively (Table-6). The FCoV, FPV, and FeLV methods agreed at 98.04%, 97.02%, and 98.81%, respectively, and the kappa values of the standard consistency test were 0.935, 0.906, and 0.976, respectively (Table-7).

**Table 6:** The clinical sensitivity and specificity of the triplex RT-qPCR.

The triplex RT-qPCR	The reported reference RT-qPCR		Total	Clinical sensitivity	Clinical specificity

	Positive	Negative			
FCoV					
Positive	206	11	217	94.50%	98.85%
Negative	12	946	958		
Total	218	957	1175		
FPV					
Positive	215	19	234	93.07%	97.99%
Negative	16	925	941		
Total	231	944	1175		
FeLV					
Positive	550	9	559	99.10%	98.55%
Negative	5	611	616		
Total	555	620	1175		

FCoV: Clinical sensitivity: 94.50% (90.63%–96.82% at 95% CI), clinical specificity: 98.85% (97.95%–99.36% at 95% CI). FPV: Clinical sensitivity: 93.07% (89.05%–95.69% at 95% CI), clinical specificity: 97.99% (96.88%–98.71% at 95% CI). FeLV: Clinical sensitivity: 99.10% (97.91%–99.61% at 95% CI), clinical specificity: 98.55% (97.26%–99.23% at 95% CI). FCoV=Feline coronavirus, FPV=Feline panleukopenia virus, FeLV=Feline leukemia virus, RT-qPCR=Reverse transcription-quantitative polymerase chain reaction, CI=Confidence interval

**Table 7:** The agreements of the triplex RT-qPCR and the reported reference RT-qPCR

Assay	FCoV		FPV		FeLV	
		
	Positive	Negative	Positive	Negative	Positive	Negative
The developed triplex RT-qPCR	217/1175 (18.47%)	958/1175 (81.53%)	234/1175 (19.91%)	941/1175 (80.09%)	559/1175 (47.57%)	616/1175 (52.43%)
The reported reference RT-qPCR	218/1175 (18.55%)	957/1175 (81.45%)	231/1175 (19.66%)	944/1175 (80.34%)	555/1175 (47.23%)	620/1175 (52.77%)
Agreement	1152/1175 (98.04%)		1140/1175 (97.02%)		1161/1175 (98.81%)	

FCoV: Positive Percent Agreement (PPA): 94.50% (90.63%–96.82% at 95% confidence interval (CI), Negative Percent Agreement (NPA): 98.85% (97.95%–99.36% at 95% CI), Overall Percent Agreement (OPA): 98.04% (97.08%–98.69% at 95% CI). FPV: Positive Percent Agreement (PPA): 93.07% (89.05%–95.69% at 95% CI), Negative Percent Agreement (NPA): 97.99% (96.88%–98.71% at 95% CI), Overall Percent Agreement (OPA): 97.02% (95.89%–97.85% at 95% CI). FeLV: Positive Percent Agreement (PPA): 99.10% (97.91%–99.61% at 95% CI), Negative Percent Agreement (NPA): 98.55% (97.26%–99.23% at 95% CI), Overall Percent Agreement (OPA): 98.81% (98.01%–99.29% at 95% CI). FCoV=Feline coronavirus, FPV=Feline panleukopenia virus, FeLV=Feline leukemia virus, RT-qPCR=Reverse transcription-quantitative polymerase chain reaction

## Discussion

FCoV [8–14], FPV [22–26], and FeLV [27–29] are prevalent worldwide, and coinfection with these viruses is common [8, 30–32]. These viruses continue to be a major factor affecting feline animals, especially kittens and multi-cat families. Therefore, it is vital to establish a technique to simultaneously detect and differentiate FCoV, FPV, and FeLV.

Multiplex RT-qPCR can simultaneously detect different viruses in a single reaction based on several pairs of specific primers and TaqMan probes, achieving high accuracy, low LOD, and rapid detection. qPCR has the advantages of avoiding contamination and quantitative detection of viral nucleic acids, and it has been widely applied in the rapid detection of etiological pathogens and accurate diagnosis of viral diseases [33]. The key to the development and application of multiplex RT-qPCR is to design specific primers and probes and to obtain optimal amplification efficiency by optimizing the annealing temperature, the concentration of primers and probes, and reaction cycles [39]. In this study, we designed three pairs of primers and probes targeting the conserved regions of the 5′ UTR of FCoV, the VP2 gene of FPV, and the pol gene of FeLV to simultaneously detect different types and strains of FCoV, FPV, and FeLV. The downstream primers were used to reverse-transcribe the RNA templates in the developed one-step triplex RT-qPCR method. Triplex RT-qPCR was developed to specifically detect FCoV, FPV, and FeLV without interference by other important feline viruses. Three standard plasmid constructs were used to generate standard curves. The amplification efficiency ranged from 100.707% to 103.756% and showed a good linear relationship with R^2^ >0.998. One-step triplex RT-qPCR showed high sensitivity with LODs of 139.904, 143.099, and 152.079 copies/reaction for FCoV, FPV, and FeLV, respectively, and excellent repeatability with 0.06%–0.87% intra-assay CVs and 0.40%–1.45% inter-assay CVs. A total of 1175 clinical samples were evaluated using the established one-step triplex RT-qPCR and the reported referenced methods [36, 38], and the agreements between these methods were 98.04%, 97.02%, and 98.81% for FCoV, FPV, and FeLV, respectively. These results demonstrated the high specificity, sensitivity, and repeatability of one-step triplex RT-qPCR and verified its feasibility in clinical applications.

The 1175 clinical samples were tested using one-step triplex RT-qPCR, and the positivity rates of FCoV, FPV, and FeLV were 18.47%, 19.91%, and 47.57%, respectively, and the coinfection rates of FCoV + FPV, FCoV + FeLV, FPV + FeLV, and FCoV + FPV + FeLV were 2.64%, 9.62%, 7.06%, and 1.96%, respectively, demonstrating that the infections of FCoV, FPV, and FeLV were serious in Guangxi province of southern China in recent years. The prevalence of FCoV, FPV, and FeLV in different provinces of China and other countries around the world have also been investigated. (1) For FCoV, fecal samples from clinically healthy cats in western Canada showed a 46.48% (86/185) positivity rate for FCoV [9]. Rectal and oropharyngeal fluid samples from cats in the Central Valley of California, USA (2020), showed a 17.65% (6/34) positivity rate for FCoV [10]. During 2015–2018, 74.6% (126/169) of samples from seven provinces of China were positive for FCoV, of which 75.7% (87/115) from feline infectious peritonitis (FIP)-suspected samples and 72.2% (39/54) from clinically healthy samples were positive for FCoV [11]. Diarrheal fecal and ascetic samples from South-west China (2017–2020) showed an 80.35% (139/173) FCoV positivity rate [13]. A total of 371 clinical samples (174 healthy cases, 81 FIP-suspected cases, and 116 diarrheal cases) from central China (2018–2021) showed 46.6% (173/371) FCoV-positive [14]. Ascitic fluid samples from FIP-suspected cats from 21 provinces in China (2019–2021) had a positivity rate of 90.83% (109/120) for FCoV [12]. (2) As for FPV. A total of 1326 samples of cats from 16 cities in China (2016–2019) were detected for FPV, FCV, FHV-1, FeLV, feline immunodeficiency virus (FIV), and FIPV, and 1060 (79.94%) cats were positive for at least one virus. FeLV, FPV, and FIPV had positivity rates of 59.6%, 19.2%, and 0.5%, respectively [22]. Rectal swabs or fecal samples from the Central and Eastern China (2018–2022) showed 35.82% (48/134) positivity rate for FPV (77.08%, 37/48) or canine parvovirus type-2 (CPV-2) (22.92%, 11/48) [23]. Egyptian cats (2020–2021) showed 35% (35/100) positivity rate for FPV and 43% (43/100) positivity rate for CPV-2 [24]. Fecal samples of 1620 diarrheal cats from Korea (2016–2019) showed 29.37% (819/1620) and 7.49% (209/1620) positivity rates for FCoV and FPV, respectively [25]. A total of 161 rectal swabs from Bangladesh (2021–2022) showed 22.9% positivity for FPV [26]. (3) For FeLV, healthy outdoor cats in Thailand (2016–2017) showed a 4.23% (11/260) positivity rate for FeLV [27]. Shelter domestic cats from California, Colorado, and Florida in the U.S. (1985–2018) showed 6.25% (19/304) positivity rate of FeLV [28]. The FeLV positivity rate was 21.19% in Italy (57/269), 20.42% in Portugal (49/240), 9.43% in Germany (30/318), and 9.35% (10/107) in France [29]. (4) Coinfection with these viruses is an important risk factor. The positivity rates for FPV, FeLV, and FCoV were 73.54% (189/257) for FPV, 23.60% (21/89) for FeLV, and 21.42% (57/266) for FCoV in Italy [8]. The 1470 necropsied cats (2010–2020) in Brazil showed a positivity rate of 26.94% (396/1470) for FeLV, 13.54% (199/1470) for FIV, and 9.12% (134/1470) for FeLV and FIV coinfection [30]. A total of 62 enteric samples from Italy (2020–2021) showed FPV and FCoV coinfection with 24.2% (15/62) [31]. All these reports for FCoV [8–14], FPV [22–26], and FeLV [27–29] indicate that these viruses are commonly prevalent worldwide, and coinfections of these viruses cannot be neglected [8, 30–32]. These findings highlight the importance and urgency of establishing multiplex RT-qPCR for the detection and differentiation of these viruses to take effective prevention and control measures.

## Conclusion

One-step triplex RT-qPCR was successfully established to detect and differentiate FCoV, FPV, and FeLV simultaneously. The assay demonstrated high sensitivity, specificity, and repeatability and can be used for rapid and accurate detection and investigation of FCoV, FPV, and FeLV. The prevalence of FCoV, FPV, and FeLV is still serious in Guangxi province, Southern China.

## Data Availability

The supplementary data can be available from the corresponding author on a reasonable request.

## Authors’ Contributions

MH: Investigation, methodology, and writing-original draft. SF: Investigation, and methodology. KS: Methodology, investigation, and writing-review and editing. YS: Data curation and analysis. FL: Supervision, validation and writing-original draft. YY: Software and data curation. ZL: Supervision and writing-review and editing. All authors have read, reviewed, and approved the final manuscript.
